# Dietary assessment and dietary guidelines across 11 European Union countries: a review from the PLAN’EAT project

**DOI:** 10.3389/fnut.2025.1699036

**Published:** 2026-01-12

**Authors:** Vittoria Aureli, Federica Grant, Alicia Aguilar-Martínez, Anke Brons, Anthony Fardet, Betty Chang, Francesca Vespa, Jana Kirschner, Ewa Kopczynska, Lajos Böröcz, Maria Jacobsen, Siranush Ghukasyan, Yannis Manios, Wencke Gwozdz, Emese Antal, Laura Rossi

**Affiliations:** 1 Departmental Faculty of Science and Technology for Sustainable Development and One Health, University Campus Bio-Medico of Rome, Rome, Italy; 2 Council for Agricultural Research and Economics - Research Centre for Food and Nutrition (CREA - Food and Nutrition), Rome, Italy; 3 Department of Agricultural, Forest and Food Sciences (DISAFA), University of Turin, Grugliasco, Italy; 4 Department of Agricultural, Environmental and Food Sciences (DiAAA), University of Molise, Campobasso, Italy; 5 Epi4Health Research Group, Faculty of Health Sciences, Universitat Oberta de Catalunya, Barcelona, Spain; 6 Public Administration and Policy, Department of Social Sciences, Wageningen University, Wageningen, Netherlands; 7 INRAE, Clermont-Auvergne University, Unité de Nutrition Humaine, Clermont-Ferrand, France; 8 European Food Information Council, Brussels, Belgium; 9 Division of Bioeconomics, Department of Earth and Environmental Sciences, Katholieke Universiteit Leuven, Leuven, Belgium; 10 European Public Health Alliance, Brussels, Belgium; 11 Institute of Sociology, Jagiellonian University, Kraków, Poland; 12 Hungarian Hospitality Employers’ Association (VIMOZS), Budapest, Hungary; 13 Department of Energy and Technology, Swedish University of Agricultural Sciences, Uppsala, Sweden; 14 TMG Think Tank for Sustainability, Berlin, Germany; 15 Department of Nutrition and Dietetics, School of Health Science and Education, Harokopio University, Athens, Greece; 16 Hellenic Mediterranean University Research Center, Institute of Agri-Food and Life Sciences, Heraklion, Greece; 17 Department of Consumer Behaviour, Nutritional Communication and Sociology, Faculty of Agricultural Sciences, Nutritional Sciences and Environmental Management, Justus-Liebig-University, Giessen, Germany; 18 Department of Management, Society and Communication, Copenhagen Business School, Copenhagen, Denmark; 19 Environmental Social Science Research Group, Budapest, Hungary; 20 Health Sciences Division, Doctoral College, Semmelweis University, Budapest, Hungary; 21 Department of Food Safety, Nutrition, and Veterinary Public Health, National Institute of Health, Rome, Italy

**Keywords:** nutritional policies, public health, sustainability, harmonized approaches, PLAN’EAT project, Europe

## Abstract

**Introduction:**

This paper presents a comparative assessment of dietary intake collection for surveillance and monitoring for adult population, Food Composition Databases (FCDBs), and Food-Based Dietary Guidelines (FBDGs) for children/adolescents, adults and the elderly population. Data and information were gathered until August 2025, concerning the 11 European countries participating in the European Horizon project PLAN’EAT “Food systems transformation toward healthy and sustainable dietary behavior.”

**Methodology:**

This assessment was conducted through questionnaires completed by PLAN’EAT partners, who collected data from scientific literature and official documents in their native languages.

**Results:**

Despite efforts such as the European Food Safety Agency’s EU Menu initiative to standardize dietary monitoring, significant inconsistencies persist in food intake assessment methodologies, database structures, and the development of dietary guidelines. These disparities hinder cross-country comparability, thereby limiting the evaluation of the effectiveness of EU-wide nutrition policies. The study identifies the aspects to be implemented in survey protocols, portion size assessment, software tools, and emphasizes the need to integrate current consumers’ eating habits when developing dietary guidelines. The importance of harmonizing data collection methods, ensuring regular national surveys, and synchronizing FBDGs updates with current consumption trends was emphasized. Key tools, such as adherence indicators and sustainability-integrated modeling approaches, were highlighted for their role in improving policy relevance and effectiveness. Diet modeling from countries like France, Germany, and the Netherlands were identified as best practices to be examined as scalable examples.

**Discussion:**

This work emphasizes the need for more coordinated EU-level actions to promote methodological consistency, enhance the sustainability and inclusivity of FBDGs, and support the development of coherent, evidence-based nutrition policies. These efforts are essential to foster healthier diets and more sustainable food systems across Europe.

## 1 Introduction

Dietary patterns are a critical entry point for improving both human health and environmental sustainability. Unhealthy dietary patterns are a key determinant of public health issues and are significantly related to the risk of developing non-communicable diseases (NCDs) (1, 2), which currently account for 80% of all premature deaths worldwide (3). This situation highlights the urgent need to address dietary risk factors and promote healthier eating habits (4). Shifting toward healthier and more sustainable dietary patterns, mostly characterized by plant-based products with less consumption of animal products, has been shown to reduce the risk of obesity, cardiovascular and cerebrovascular diseases, and all-cause of mortality together with improving overall human health outcomes (5–8). At the same time, these healthier dietary patterns can address the environmental impact of current food consumption. Dietary patterns characterized by lower consumption of animal products (especially red and processed meat and dairy), and nutritionally poor foods, typically high in added fats, sugar, and salt, alongside higher intake of plant-based foods, such as fruits, vegetables, legumes and nuts, have been considered more environmentally sustainable (9–12). Current eating habits, which are high in animal-based foods and nutritionally poor products, contribute to the increased greenhouse gas (GHG) emissions, deforestation, biodiversity loss, and water depletion, further exacerbating climate change and environmental degradation (13). For this reason, switching dietary patterns toward plant-based diets, reducing the intake of animal products and nutritionally poor foods, should be encouraged since this would lead to a significant reduction of the environmental impact (14–16), with a potential mitigation estimated as 0.7–8 Gt CO_2_ equivalents per year by 2050 (17).

In addition to this scientific evidence, several European Union (EU) countries have already implemented national Food and Nutrition Strategies aimed at improving public health and promoting sustainable food systems. For example, within PLAN’EAT project, Germany has implemented the “Good Food for Germany” (18) programme, while France has established both the “Programme National Nutrition Santé” (19) and the “Programme National pour l’Alimentation” (20). These strategies outline not only clear political objectives, such as reducing diet-related diseases and encouraging healthier and more sustainable eating habits but also include concrete policy measures to achieve these goals. Dietary habits across EU countries vary significantly due to different cultural traditions, food systems and socioeconomic factors (21–23). To design effective public health policies and regulations together with practices that promote a shift toward healthier and more sustainable dietary patterns, it is essential to recognize and account for these differences. Significant changes in dietary behaviors can be achieved by addressing determinants at multiple levels of influence, including individual practices, food environments, and policy frameworks (24–26). In line with this question, recent evidence from global food system analyses highlights that transforming these systems is fundamental to improve population healthiness and sustainability. By integrating scientific evidence with coordinated policies across agriculture, health, and economic sectors, countries can create supportive food environments that facilitate healthier dietary choices (27).

To accomplish these objectives, reliable and standardized dietary intake data are essential for evaluating the nutritional and health status of populations, monitoring trends, and informing policymakers on the implementation status of targeted nutritional interventions (28). However, the methodologies and tools used to evaluate and monitor dietary patterns differ across EU countries, leading to challenges in data comparability and policy effectiveness (29). Despite ongoing efforts to harmonize methodologies at the European level, such as the European Food Safety Authority (EFSA) EU Menu initiative launched in 2014 (30), significant discrepancies persist regarding the timing of regional or national dietary surveys, the food composition databases used at national level, and the structure and updating of Food-Based Dietary Guidelines (FBDGs) across EU member states. Linking FBDGs to national food-consumption data, collected through surveys and dietary assessments, can enable policymakers to observe population adherence to dietary recommendations, identify dietary gaps, and tailor interventions to improve healthiness eating patterns (31).

Dietary guidelines represent a cornerstone of nutrition policies, translating scientific evidence into clear and actionable recommendations for healthy eating (32). By defining optimal intake levels for key food groups and nutrients, the guidelines inform public health campaigns, school meal standards, and food procurement regulations (33). Ongoing monitoring of consumption trends and of the population’s nutritional and wellbeing status, in turn, should inform periodic updates of the guidelines. This approach aims to ensure that the guidelines remain evidence-based, culturally appropriate, and capable of allowing measurable improvements in public health. Therefore, considerable coordination, investment in methodological alignment, and the development of common frameworks are required to improve dietary monitoring and policy integration across Europe.

To address these challenges, the present work focuses on three areas due to their pivotal role in shaping evidence-based nutrition policies and enabling cross-country comparisons of dietary consumption levels and food behaviors: food consumption, food composition databases and FBDGs. By identifying key discrepancies and gaps, while highlighting best practices that can be replicated, this work provides valuable insights to support the development of more coherent and effective food policies across EU. In the context of the EU Horizon PLAN’EAT^1^ Project a scoping review was conducted in the 11 participating countries (Belgium, France, Germany, Greece, Hungary, Ireland, Italy, Poland, the Netherlands, Spain, and Sweden) to assess similarities and differences across countries focusing on three key nutrition-related issues: (1) protocols and frequency of dietary intake data collection, (2) food composition databases employed at the national level, and (3) structure and content of national FBDGs, with particular emphasis on methodology and revision processes, dietary recommendations, target population groups covered, behavioral recommendations, and sustainability aspects.

Therefore, the main research questions of this study are: (i) how do national approaches to dietary data collection, food composition databases, and FBDGs differ across EU countries? and (ii) what actionable recommendations could be made to address these differences and foster coordinated, sustainable nutrition policymaking at both national and EU levels? Rather than proposing a one-size-fits-all model, this study aims to promote comparability while respecting national contexts. This approach may enable EU institutions and national authorities to better align health and sustainability objectives. A systematic overview of current practices is important to assess progress toward shared EU goals, identify replicable and scalable best practices, and guide future policy actions. This work is intended to be relevant for (i) EU policymakers working toward harmonized and monitoring food policy frameworks; (ii) public health and nutrition authorities aiming to strengthen national systems and contribute to a broader European agenda; (iii) researchers and academia conducting comparative studies in nutrition and public health.

## 2 Assessment of dietary intake and guidelines’ options and implications

### 2.1 Methodological framework

A structured discussion with 16 PLAN’EAT project partners, representing 16 consortium organizations, was conducted to assess national dietary intake data and guideline options across the 11 participating countries, meaning Belgium, France, Germany, Greece, Hungary, Ireland, Italy, Poland, the Netherlands, Spain, and Sweden. An informative questionnaire was designed and organized into four sections (Supplementary material):

- Food composition databases (7 items, 10 questions),
- Food Based Dietary Guidelines (6 items, 8 questions),
- Food consumption (what people eat) (6 items, 9 questions),
- Eating habits (how people eat) (13 items, 35 questions).

The draft questionnaire was first shared with partners for feedback, before completion. In a second phase, partners completed the questionnaire supported by scientific literature and official documents in their native language. When necessary, they sought input from national experts to ensure accuracy and contextual relevance.

To ensure consistency and accuracy, the PLAN’EAT project coordination team at the Council for Agricultural Research and Economics (CREA) implemented a double-check process. This involved reviewing completed questionnaires and discussing with partners to verify sources and resolve implementation issues. When gaps or missing information were identified, and when documents did not include the required details, the CREA team directly searched for relevant national information. Partners were then asked to review the newly added information to confirm the accuracy and completeness. Data were analyzed both within each country and across countries to identify commonalities, gaps, and differences.

Data and information were gathered from September 2022 to August 2025.

### 2.2 Dietary intake assessment

Dietary intake assessment is fundamental for understanding what people eat, evaluating dietary patterns, nutrient adequacy, and identifying diet-related health risks (34). Tracking changes over time and across populations enables detection of emerging nutritional issues, monitoring of policy effectiveness, and research into diet–health relationships (35).

Within PLAN’EAT, dietary intake assessment comprises two components:

- Collection of food consumption data—records the types and quantities of foods consumed.
- Collection of food composition database (36)—provides nutrient profiles for these foods.

Combining these two datasets, consumption data might be converted into nutrient intake estimates, enabling assessment of dietary adequacy and identification of nutritional deficiencies. This integration could support the development of targeted nutrition policies, assessment of dietary recommendations, and harmonizing cross-country comparisons (37).

#### 2.2.1 Food consumption data measurement

Food consumption data measurement is fundamental to characterize dietary patterns, tracking trends in dietary behaviors, and identifying potential diet-related health risks.

Since 2005, EFSA has set up strategies to harmonize data collection and created the EFSA Comprehensive European Food Consumption Database in collaboration with national experts (38). In 2011, EFSA launched the EU Menu Project to standardize the collection of accurate, harmonized, and detailed individual-level food consumption data (30).

Table 1 summarizes the national dietary surveys for adult populations in the PLAN’EAT countries as listed in the EFSA database (38). However, updated, surveys from Ireland (39) and the Netherlands (40, 41) are available on national websites but have not yet been included in the EFSA database. Therefore, the following analysis focuses on surveys listed in the EFSA database.

**TABLE 1:** List of dietary national surveys for adult population of European countries participating in the PLAN’EAT project included in the EFSA database.

Country	Food consumption DataBase	Responsible body	Years of collection	Methodology	References	EU menu
Belgium	Belgian national food consumption survey in children, adolescents and adults (BNFCS2014)	Sciensano (Scientific Institute of Public Health)	2014–2015	24-h dietary recall	(42)	Yes
France	The French national dietary survey (INCA3, 2014-2015)	National Agency for Food, Environmental and Occupational Health and Safety (ANSES)	2014–2015	24-h dietary recall	(43)	Yes
Germany	German National Nutrition Survey (NVSII)	Max Rubner Institute (MRI)	2005–2008	24-h dietary recall	(44)	No
Greece	The EFSA-funded collection of dietary and related data in the general population aged 10-74 years in Greece	Hellenic Health Foundation (HHF) and the Hellenic Ministry of Health and the Center for Disease Control	2014–2015	24-h dietary recall	(45)	Yes
Hungary	Hungarian national food consumption survey	National Food Chain Safety Office	2018–2020	24-h dietary recall	(46)	Yes
Ireland	National Adult Nutrition Survey (NANS 2012)	Irish Universities Nutrition Alliance (IUNA)	2008–2010	Food record	(47)	No
Italy	Italian national dietary survey on adult population from 10 up to 74 years old IV SCAI ADULT 2018-2020	CREA-Food and Nutrition Research Center	2018–2020	24-h dietary recall	(48)	Yes
The Netherlands	Dutch National Food Consumption Survey 2012-2016 (DNFCS)	National Institute for Public Health and the Environment (RIVM)	2012–2016	24-h dietary recall	(49)	Yes
Poland	National Dietary Survey on the adult population	National Institute of Public Health PZH – National Research Institute	2019–2020	24-h dietary recall	(50)	Yes
Spain	Spanish National dietary survey in adults, elderly and pregnant women	Spanish Agency for Food Safety and Nutrition	2013	24-h dietary recall	(51)	Yes
Sweden	Swedish National Dietary Survey - Riksmaten adults 2010-11	Swedish National Food Agency (Livsmedelsverket)	2010–2011	Web-based dietary record	(52)	No

Information reported: the name of the database; the responsible for conducting the national dietary survey; years of collection; methodology applied; reference to the national survey; the application of the EU Menu methodology.

Important considerations:

- Survey frequency varies: Hungary, Italy, Poland carried out recent dietary surveys, whereas Germany, Ireland, Sweden rely on older data, less reflective of current dietary patterns.
- Dietary assessment methods differ for Ireland (food record) and Sweden (web-based dietary record) since the national dietary surveys were carried out prior to the EU Menu methodology release.
- Institutional responsibility varies in most countries, national public health institutes or food safety agencies manage data collection. In Ireland and Italy, academic or research institutions carry out this work (Table 1).

#### 2.2.2 Food composition databases

Food composition databases (FCDBs) are essential for dietary assessment, providing standardized data on the nutritional profiles of food products, including macro- and micronutrients (53). All 11 PLAN’EAT countries maintain a national FCDB, except for Ireland, which uses the UK database adapted to the Irish market (Table 2). The frequency of updates varies: while most countries revise their database regularly, Hungary and Greece have not updated theirs since 2005 and 2004, respectively. In some cases, secondary FCDBs are maintained alongside the official versions. For example, Greece has *The Hellenic Food Thesaurus – Health* in addition to the Greek National Food Composition Database, while Italy supplements CREA’s “Food Composition Tables” with the European Institute of Oncology (IEO) database for epidemiological studies.

**TABLE 2:** List of national food composition databases of European countries participating in the PLAN’EAT project.

Country	Food composition DataBase	Responsible body	Last update	Link
Belgium	Belgian NUBEL database	The Nubel foundation	2022	The Nubel Food Planner
France	French food composition database	Ciqual (Food Quality Information Center) under the supervision of the authority of the National Agency for Food, Environmental and Occupational Health and Safety (ANSES)	2020	Table de composition nutritionnelle des aliments Ciqual
Germany	Bundeslebensmittelschlüssel (BLS)	Max Rubner Institute (MRI)	2014	Bundeslebensmittelschlüssel - BLS
Greece	Greek national food composition database (FCD)	Dr. Antonia Trichopoulou, Director of the World Health Organization, Collaborating Centre of Nutrition, University of Athens	2004	ΠINAKAΣTPOΦIMΩN
	The Hellenic Food Thesaurus (HelTH)	Dr. Maria Kapsokefalou, Professor in Human Nutrition at the Agricultural University of Athens	2020 (not yet completed)	Βάση δεδομένων συσκευασμένων τροφίμων
Hungary	Hungarian food composition database	National Institute of Pharmacy and Nutrition (OGYÉI)	2005	New Nutrient Tables. Medicina Könyvkiadó Rt., Budapest (54)
Ireland*	UK Food Composition Database	Public Health England (PHE)	2021	Composition of foods integrated dataset (CoFID)
Italy	Food composition tables	CREA-Food and Nutrition Research Center	2019	AlimentiNUTrizione - Tabelle Composizione Alimenti
	Food composition database for epidemiological studies in Italy	Epidemiological Division of The European Oncology Institute of Milan (IEO)	2022	BDA – Banca Dati di composizione degli Alimenti per studi Epidemiologici in Italia
The Netherlands	Dutch Nutrient Database “NEVO”	National Institute for Public Health and the Environment (RIVM)	2023	Dutch Nutrient Database “NEVO”
Poland	Polish food composition database	National Institute of Public Health PZH – National Research Institute	2017	Tabele wartości odżywczej produktów spożywczych i potraw
Spain	Spanish Food Composition Database BDECA	Ministry of Science and Innovation and the Spanish Agency for Food Safety and Nutrition of the Ministry of Health, Social Services and Equality	2010	Base de Datos BEDCA
Sweden	Swedish Food Composition Database	Swedish National Food Agency (Livsmedelsverket)	2025	Search for nutrients - Livsmedelsverket

Information reported: the name of the database; the responsible for developing and updating the database; last update of the data; link to the database repository. * Ireland uses the UK Food Composition Database at the national level, adapting it to the Irish market when necessary.

FCDBs are compiled drawing on various methodologies: (1) chemical analysis of foods, entailing the identification, separation, and quantification of food components to determine their composition; (2) incorporation of data from other national datasets; and (3) supplementary information from scientific literature, branded foods, and food labels. At the European level, harmonization is supported by the European Food Information Resource (EuroFIR), which standardizes data through the FoodEXplorer system, facilitating cross-country comparisons (55). Most PLAN’EAT countries are represented in the EuroFIR (56), except for Hungary.

When interpreting FCDBs data, several limitations should be noted. Many national databases are not fully up to date and may not cover the growing variety of food products on the market, including different brands, traditional foods, and numerous macro- and micronutrients and trace elements. Data on environmental impact indicators such as land use, water footprint, and carbon footprint, as well as on processing levels and additives, are often absent.

The type of institution responsible for data collection and updating also varies: while health authorities manage most FCDBs, in Belgium this responsibility lies with a foundation, in Greece with universities, and in Italy with public research bodies not working in the health sector.

### 2.3 Food-Based Dietary Guidelines

All European countries have national dietary guidelines designed to improve nutritional status of their populations with different requirements. These FBDGs are widely acknowledged as essential policy instruments for improving public health and preventing diet-related chronic diseases (57, 58). The comparative analysis reveals both shared principles and substantial differences in their development methodologies, scope and implementation.

#### 2.3.1 FBDGs development methodology and updates

In most countries, FBDGs are developed through scientific evidence reviews, but the scope and complexity of these reviews vary. Germany, the Netherlands and France apply diet optimization modeling to identify combinations of foods that meet nutritional requirements while respecting specific constraints such as environmental impacts or alignment with current consumption patterns (57, 59–62). Germany and the Netherlands integrate both health and environmental considerations, while France focuses solely on health constraints. However, all three countries took into account the population’s habitual dietary patterns to produce recommendations aligned with current eating habits. Ireland used a modeling approach for FBDGs targeted specifically at children aged 1–5 years based on health criteria (63).

This modeling approach allows simultaneous analysis of different aspects of a diet, such as energy intake, affordability and environmental impacts, and supports different objectives while maintaining nutritional adequacy. However, while this methodology cannot account for all cultural habits, including current dietary patterns, it ensures that outputs are realistic and applicable in the national context.

The frequency of FBDGs updates vary. Most of the countries have revised their guidelines in recent years, whereas Ireland and Greece reported the last update in 2016 and 2017, respectively. The institutions responsible for developing and updating FBDGs also differ: in most cases, these are national public health institutes or food safety agencies, but in Greece the responsibility is shared between academic institutions and the Ministry of Health and Education; in Italy, it lies with public research organizations outside the health sector; in Hungary, with professional dietetic associations; and in the Netherlands and Germany, with dedicated nutrition centers or societies (Table 3).

**TABLE 3:** List of national FBDGs of European countries participating in the PLAN’EAT project.

Country	Food-Based Dietary Guidelines	Responsible body	Update	References
Belgium	Belgian dietary guidelines	Superior Health Council	2019	(63)
France	French dietary guidelines, PNNS 4 (the Programme National Nutrition Santé n°4)	National Agency for Food, Environmental and Occupational Health and Safety (ANSES)	2019	(64)
Germany	Food-Based Dietary Guidelines for Germany	German Nutrition Society (DGE)	2024	(65, 66)
Greece	The “National Nutrition Guide for Greek” (NDGGR)	Representatives of academia, Ministry of Health and Ministry of Education and Culture	2017	(67)
Hungary	SmartPlate	Hungarian Dietetic Association and the Hungarian Academy of Sciences	2021	(68)
Ireland	The Healthy Eating Guidelines	Department of Health (Health Service Executive)	2016	(69)
Italy	The Italian dietary guidelines	CREA-Food and Nutrition Research Center	2018	(70)
The Netherlands	Wheel of Five	Netherlands Nutrition Centre (NNC)	2024	(58, 71)
Poland	Plate of Healthy Eating	National Institute of Public Health PSH—National Research Institute—National Institute of Hygiene (NIZP-PZH NIZP PZH–PIB)	2021	(72, 73)
Spain	Spanish dietary guidelines	Agencia Española Seguridad Alimentaria y Nutrición (AESAN)	2022	(74)
Sweden	Find your way–Eating habits and dietary guidelines	Swedish National Food Agency (Livsmedelsverket)	2025	(75)

Information reported: the name of FBDGs; the responsible for developing FBDGs; more recent update; reference to the document (s).

#### 2.3.2 Dietary recommendations

The approaches used to develop FBDGs dietary recommendations range from general qualitative dietary advice to detailed quantitative guidelines specifying frequency and portion sizes. Most of the 11 PLAN’EAT countries provide portion sizes for several food groups. In Hungary, Ireland, and the Netherlands, these portion sizes are expressed in household measures (e.g., cups, handfuls) to improve practical understanding. Sweden stands out for providing qualitative recommendations without quantitative guidance.

Analysis of recommendations for the adult population (Table 4) shows broad alignment with the World Health Organization’s recommendations on fruit and vegetables intake (77). Most countries recommend at least five servings per day, with Greece and Ireland promoting seven servings and emphasizing diversity. Whole-grain products are consistently recommended as the preferred source of carbohydrates. Legumes are promoted as a nutritious, plant-based source in all the countries.

**TABLE 4:** FBDGs recommendations for common food groups targeting the adult population in the European countries participating in the PLAN’EAT project.

Food groups	Belgium	France	Germany	Greece	Hungary	Ireland	Italy	The Netherlands	Poland	Spain	Sweden
**Fruit and vegetables**	250 g of fruit and 300 g of vegetables/day	5 fruit and vegetables/day	5 potions/day SP: 110 g	3 servings of fruit and 4 servings of vegetables/day SP vegetables: 150–200 g raw/cooked and fruit: 120–200 g	At least 5 portions/day: 3–4 portions of vegetables/1–2 portions of fruit (at least 1 portion should be fresh/freshly cut) SP: 1 large pepper, tomato, 1 large apple or peach or 1 medium bowl of lettuce or 80 g dry or 120 g fresh/frozen pulses or 1 cup of berries or 2 dl smoothie	5–7 servings/day SP: 1 medium size fruit, 2 small fruits, 1/2 cup cooked vegetables, 1 bowl salad	3 of fruit and 2 1/2 of vegetables times/day SP fruit: 150 g and vegetables: 200 g	250 g of vegetables and 200 g of fruit/day SP vegetables: 50 g and fruit: 100 g	400 g/day 1/4 of plate is fruit; 3/4 of plate is vegetables	3 servings of fruit at least and 2 servings of vegetables/day SP vegetables: 150–200 g and fruit: 120–200 g	500 g/day vegetables, fruit and berries
**Legumes**	Included in the vegetables consumption Consume legumes every week	At least twice a week; can replace meat but to be combined with cereals	1 portion/week SP: 125 g (ready to eat)	3 servings/week SP: 150–200 g of cooked legumes	At least once a week (included in the vegetables consumption)	2 servings/day* SP: 3/4 cups	3 times/week SP: dry legumes: 50 g; fresh legumes: 150 g	2–3 servings/week SP: 60 g	2–3 times/week SP: 50 g; dry portion	4 servings/week SP: 50–60 g raw	Eat beans, peas and lentils often—preferably every day.
**Nuts**	15–25 g/day	A handful/day	1 portion/day (together with seeds) SP:25 g	1–2 servings/day SP: 18 almonds, 6 whole walnuts, 3 tablespoons of sunflower seeds	2–3 times/week SP: small handfuls of nuts, unsalted almonds, hazelnuts, oilseeds such as pumpkin seeds	2 servings/day* SP: 40 g	2 times/week SP: 30 g	15–25 g/day	30/40 g/day	3 or more servings/week SP: 20–30 g	Eat two to three tablespoons of unsalted nuts a day
**Grain-based foods** **Whole grains**	At least 125 g/day of whole-grains	At least one portion of whole-grain starchy food/day	5 portions/day SP:60 g (one lice of bread, one serving of cereal flakes, uncooked pasta/rice) At least 1/3 should be whole grain	5–8 serving of refined and whole-grain cereals/day SP: 1 slice or 30 gr of bread, 1/2 cup of cooked pasta or rice, 1/2 of breakfast cereals, 1 medium potato: 120–150 g cooked	3 times/day at least one portion out of three should be whole-grain SP: 1 piece of sweet bread dough or 1 medium slice bread/brioche bread or 12 tablespoons (200 g) cooked pasta/rice or 3 tablespoons of breakfast cereals	3–5 servings/day SP: 1 cup cooked rice, pasta, noodles or cous cous, 2 thin slices whole meal bread Enjoy whole grains at each meal	Bread 3 ^1/2^ times/day SP 50 g; Pasta, rice, etc. 1 ^1/2^ times/day SP: 80 g Prefer whole-grain products	Bread 4–8 slides/day; SP: 35 g 3–5 servings/day of cereal products and potatoes; SP: tablespoon of cereals: 50 g; medium potato: 70 g At least half of whole-grain grain products every week.	90 g 3 times/day of whole grain cereals	3–6 servings/day SP: 40–60 g bread, 60–80 g pasta, rice Prefer whole-grain products	At least 90 grams of whole grains per day,
**Meat** **Red meat** **Processed meat** **White meat**	Maximum 300 g/week of red meat Maximum 30 g/week of processed meat 1–3 times/week (including eggs/meat substitutes) Limit the consumption of red meat, especially processed meat. Red meat can be replaced by e.g. legumes, fish, eggs or poultry	Prioritize poultry and limit red meat to 500 g/week Charcuterie: limit to 150 g/week	1–2 portions of beef, pork, poultry/week SP: 120 g 2 portions of sausages/week SP:30 g Do not consume more than 300 g/week of meat and sausage	1 serving of lean red meat/week SP: 120–150 g of cooked meat 1–2 servings of white meat/week SP: 120–150 g of cooked meat. Processed meat: as few as possible.	Choose lean variants more often. Consume not more than 350–500 g/week of cooked/steamed/ fried red meat (e.g., beef, pork). Processed meat only occasionally, in small amounts.	2 servings/day* SP: 50–75 g (beef, lamb, pork, poultry) Limit processed salty meats	Once/week of red meat SP: 100 g 2 times/week of white meat SP: 100 g Limit the consumption of processed meat (occasionally consumption)	Max 500 g/week of which max 300 g of red meat SP: 100 g/day excluding processed meat and eggs Limit the consumption of red and processed meat.	Not more than 350–500 g of red meat and processed meat/week For the white meat: choose lean poultry meat (e.g., chicken, turkey) without the skin	0–3 servings/week for meat, preferring the white meat SP:100-125 g For processed meat: reduce or even avoid consumption	No more than 400–500 grams of raw meat. (no more than 350 g cooked) Only a small part of it should be cured meat.
**Fish**	1–2 times/week (oily fish once/week)	2 times/week of which once fatty fish	1–2 portions/week SP: 120 g	2–3 servings of fish and seafood/week SP: 150 g of cooked fish or seafood	At least once/week Prefer local fish (e.g., trout, catfish, bighead carp).	2 servings/day * SP: 100 g	2 times/week SP: 150 g	1 serving/week, preferably fatty fish SP:100 g	2 times/week SP: 100–150 g	3 servings/week SP: 125–150 g	2–3 times/week
**Dairy products** **Milk** **Yogurt** **Cheese**	250–500 ml/day of dairy	2 times/day of dairy	2 portions/day Milk SP: 250 g Yogurt SP: 150 g Cheese SP: 30 g	2 servings/day SP: 250 ml of milk, 200 g of yogurt, 30 g of seasoned cheese, 60 g of fresh cheese	Every day SP: 200 ml milk/yogurt/kefir or 50 g cottage cheese or 30 g cheese	3 servings/day SP: 200 ml of milk, 125 g of yogurt, 25 g of cheese	Milk/yogurt 3 times/day SP: 125 ml/125 g Cheese 3 times/day SP:100 g fresh; 30 g seasoned	2–4 servings/day of milk and dairy SP:150 g Cheese SP: 40 g/day	Prefer low-fat products	3 servings/day SP: milk: 200–250 ml; fresh cheese 85–125 g; seasoned cheese 40–60 g; 125 g yogurt	Milk and yogurt every day; cheese can replace a small share of milk; refer low fat products.
**Eggs**	1–3 times/week with poultry/meat substitutes	NA	1 egg/week SP:60 g	At least 4 times/week	Replace meat with other protein sources, including eggs	2 servings/day*	3 times/week SP: 50 g	2–3 times/week SP: 50 g	Good source of proteins and other nutrients, maximum 7 eggs a week	4 times/week SP: 50–60 g	Good alternative to meat
**Sugary foods**	Consume as few drinks with added sugars as possible and choose water instead	Limit the foods rich in sugar	Avoid products with sugar	Limit added-sugar products/sugar	Limit added-sugar products/sugar	NOT every day	Occasionally	Outside the wheel of five	Reduce the consumption	Avoid the consumption	Limit sugary products
**Oils/fats**	Prefer non-tropical oils, spreadable fats and soft or liquid cooking fats	Prefer olive, walnut, rapeseed oil	1 tablespoon/day of vegetable oils 1 tablespoon/day of butter/margarine SP: 10 g	Prefer olive oil (4–5) times/day; SP: 15 ml	Less fat for cooking, prefer oils	Rapeseed, olive, canola, sunflower or corn oils (in a very small amount)	3 times/day SP: 10 ml	Spreadable and cooking fats (not specified) SP: 35–65 g	Prefer vegetable oils, reduce animal fat	Olive oil in every meal (10 ml)	Prefer vegetable oils

Food groups considered: 1) Fruit and vegetables, 2) Legumes, 3) Nuts, 4) Grain-based foods; 4a) Whole-grains, 5) Meat, 5a) Red meat, 5b) Processed meat, 5c) White meat, 6) Fish, 7) Dairy products; 7a) Milk, 7b) Yogurt, 7c) Cheese, 8) Eggs, 9) Sugary foods, 10) Oils/fats. For each food group, the quantity of the standard portion and the number of servings recommended were reported, when available. *For Ireland, the frequency of 2 servings/day considers together all the food groups indicated with the asterisk. SP, standard portion; NA, not available.

For meat, most countries recommend limiting red meat to 300–500 g per week, promoting white meat as a healthier option and discouraging processed meat consumption. Fish recommendations are generally consistent, with advice to consume one to two servings per week; Belgium, France, Germany, and the Netherlands highlight the health benefits of fatty fish. Sugary foods and sweetened beverages are restricted in all the guidelines, with emphasis on moderation in daily sugar intake.

Despite these similarities, differences remain. Portion size definitions vary widely across countries, together with the frequency of recommendations for certain food groups, complicating comparability across countries. In addition, food categories do not always include the same products, e.g., legumes can be placed with fruit and vegetables, in protein-source group, or in an own category. In some countries, environmental considerations are explicit in the recommendations, while in others they are only indirectly addressed through promotion of plant-based foods.

Although the International Agency for Research on Cancer (IARC) has classified alcohol as a Group 1 carcinogen (78) and its consumption should therefore be avoided, an analysis was conducted to explore how the PLAN’EAT countries address this issue within their FBDGs. Germany, Italy, and the Netherlands explicitly advise against alcohol consumption, and France, Germany, Ireland, Italy, and the Netherlands provide quantitative limits. Poland, Spain, and Sweden address alcohol outside the FBDGs, with Spain and Sweden explicitly discouraging its consumption (79–81).

#### 2.3.3 Target groups

Within general national FBDGs, some PLAN’EAT countries provide specific recommendations for population subgroups, while others have developed separate targeted guidelines. Greece, Ireland, Italy, the Netherlands, and Sweden include tailored recommendations for women at key life stages such as pregnancy, breastfeeding, and menopause, although the latter is not covered in Ireland, the Netherlands and Sweden (Table 5). France includes these recommendations outside the guidelines.

**TABLE 5:** FBDGs recommendations of the PLAN’EAT countries regarding different target groups: pregnancy, breastfeeding and menopause conditions; infant, children and adolescents; the elderly.

Country	Pregnancy, breastfeeding and menopause inside/outside the general FBDGs	Infants, children, and adolescents inside/outside the general FBDGs	The elderly inside/outside the general FBDGs
France	Outside (82)	Outside (82)	Outside (82)
Greece	Inside (83)	Inside (84)	Inside (85)
Hungary	NA*	Inside (4-17 years old) (69)	Outside (86)
Ireland	Inside (menopause not included) (87, 88)	Outside (1-5 years old) (63)	Outside (70, 89)
Italy	Inside (71)	Inside (71)	Inside (71)
The Netherlands	Inside (menopause not included) (90, 91)	Inside (92)	Inside (92)
Sweden	Inside (menopause not included) (93, 94)	Inside (0-2 years old) (95)	Inside (96)

*NA: not available information for the target group recommendations. For each target group the inclusion of the exclusion of the related recommendations within the guidelines are reported. Outside: exclusion; inside: inclusion.

Age-specific guidance for infants, children, and adolescents is included in the FBDGs of Greece, Hungary, Italy, the Netherlands, and Sweden, although France and Ireland provide these recommendations separately (Table 5). Guidance for the elderly is present in the general FBDGs of Greece, Italy, the Netherlands, and Sweden emphasizing muscle mass preservation, adequate protein intake, and prevention of age-related health complications (Table 5). France, Hungary and Ireland address this outside their general FBDGs.

Some countries extend beyond conventional population groups to address cultural diversity and dietary preferences. The Dutch FBDGs include tailored recommendations e.g., for vegetarians, vegans and individuals following non-Western dietary traditions, particularly those among common immigrant populations such as Turkish, Moroccan, and Surinamese communities (92). The Swedish FBDGs provide general advice for the overall population, along with specific recommendations for children, on how to follow a vegetarian or vegan diets (97, 98).

#### 2.3.4 Physical activity and other behavioral advice

All the countries, except from Belgium, provide physical activity recommendations within the FBDGs, though their integration and specificity vary. In Ireland (99) and the Netherlands (100), these appear as supplementary guidance. Germany promotes physical activity through general advice, such as stay active (67), while France and Poland offer more specific and quantitative recommendations, such as daily walking or stair use (65, 73). Greece, Italy, Spain, and Sweden provide detailed guidelines on duration and intensity by population group (71, 75, 101, 102). Hungary emphasizes lifelong adaptability in physical activity. Poland and Spain also include step count targets, such as 10,000 steps per day, aligning with the World Health Organization’s guidance (103).

Beyond physical activity, all PLAN’EAT countries include broader lifestyle recommendations. Common recommendations include maintaining a balanced and varied diet (Germany and Italy), establishing a structured meal routine (Greece, Hungary, Ireland, Italy, and Poland), avoiding digital distractions during meals (France, Germany, Greece, and Italy), and preparing home-cooked meals with fresh ingredients (Belgium, France, Germany, Greece, Hungary, Ireland, and Italy). Some countries promote informed food choices through nutrition labeling and encourage local, organic, and seasonal foods (France, Hungary, Italy, the Netherlands, Spain, and Sweden). Food safety and waste reduction measures including meal planning and the use of shopping lists are also addressed in Hungary, Italy, the Netherlands, Spain and Sweden. Ireland highlights the importance of adequate sleep and time outdoors, while Germany emphasizes mindful eating.

#### 2.3.5 Sustainability

Nine of the eleven PLAN’EAT countries incorporate sustainability into their FBDGs. The Netherlands, and Germany, have developed FBDGs using a mathematical optimization modeling that integrates sustainable criteria alongside health criteria. Italy, Poland, Sweden, and Spain put a strong emphasis on sustainability: Italy dedicates an entire chapter to plant-based food consumption, promoting eco-friendly cooking practices, seasonal, and locally sourced foods while limiting red and processed meats. Poland includes specific recommendations on how to decrease the environmental impact adopting diets that are more plant-based, local, and that include unprocessed products. Sweden provides detailed environmental impact information for each food group, recommending eco-labeled and eco-friendly choices. Spain advocates local, fair-trade, and low environmental-impact foods throughout its guidelines.

Belgium, France and Hungary do not have dedicated sections on sustainability, but encourage plant-based diets, reduced meat consumption, and the use of local, seasonal, and organic foods.

## 3 Actionable recommendations

The in-depth cross-country comparison of dietary assessments, across the 11 European countries, identified several priority areas where methodological alignment and institutional strengthening could substantially enhance the development of coherent, evidence base of nutrition policies. Harmonizing survey protocols as recommended by EFSA and using shared platforms would improve comparability of food consumption data, enabling timely detection of dietary trends and nutrient deficiencies (104). Similarly, standardizing the compilation, updating, and coding of nutrient values across national FCDBs would help to address persistent gaps and inconsistencies in nutrient intake estimates (105).

In parallel, the adoption of quantitative modeling approaches that incorporate both health outcomes and environmental impacts could transform FBDGs from static sets of recommendations into adaptable framework capable of integrating new evidence (106, 107). Following EFSA recommendations, regular guideline updates, transparent documentation of decision-making processes, and the seamless inclusion of subgroup-specific recommendations (e.g., for pregnancy and older age) would improve the clarity, relevance, and credibility of national guidelines (58).

Together, these measures could create a cyclical policy feedback mechanism: harmonized data collection would strengthen policy modeling; improved models would refine dietary recommendations; and updated guidelines would inform targeted public health interventions, which could then be evaluated using the same harmonized data systems (108). This integrated approach would not only improve the scientific basis of EU nutrition policies but also empower governments, researchers, and health professionals to work collaboratively using a shared, high-quality evidence base to promote healthier and more sustainable diets.

Based on the comparative analysis of the 11 PLAN’EAT countries, the following actionable recommendations are proposed. However, to move from recommendations to implementation, it is essential to identify concrete mechanisms, responsible actors, and enabling resources:

- *Standardization of Dietary Intake Assessment:* (1) expand the EU Menu initiative to ensure uniformity in methodology, consistent reporting formats, and regular updates across Member States; (2) Provide financial and/or technical support at EU level to help countries with outdated or incomplete surveys conduct new and methodologically aligned dietary assessments; (3) encourage the standardized methodology suggested by EFSA to compare data across country; and (4) establish a standard maximum interval (e.g., every 5 or 10 years, while the observed mean interval among PLAN’EAT countries is 10 years, with a minimum of 4 years in Spain and a maximum of 19 years in Poland, with several countries having their most recent surveys for adult population conducted before 2010 for national dietary surveys) to ensure data remain updated and useful for monitoring changes in eating patterns. This would require coordinated funding calls under different EU projects, technical training workshops for national survey teams, and an EFSA’s platform to monitor survey standardization, implementation and compliance.
- *Harmonize Food Composition Databases:* (1) link EFSA’s consumption data with the EuroFIR food composition database to create a unified, integrated resource. A key current limitation is the lack of integration between the LanguaL descriptor system and EFSA’s food consumption data; (2) incorporate secondary data sources into national FCDBs to address gaps, particularly for region-specific foods and bioactive compounds; (3) Develop standardized protocols for food composition analysis across countries and establish mechanisms for rapid inclusion of new food products, ensuring that databases accurately reflect current market availability and consumption patterns. This could be carried out by setting up a permanent European Data Hub, with shared digital infrastructure for data exchange, and national contact points responsible for real-time updates on new products available on the European market.
- *Harmonization of FBDGs at the European level:* (1) develop a common methodological framework defining the types of evidence and modeling approaches to be used in formulating FBDGs; (2) Ensure inclusivity by tailoring recommendations to the needs of specific population groups, including children, the elderly, individuals with particular physiological conditions, and diverse socio-economic backgrounds; (3) implement a regular guideline revision cycle—ideally every 5–10 years—to keep FBDGs aligned with the latest scientific evidence and societal trends. This could be facilitated through an European taskforce under EFSA coordination, supported by structured experts’ consultations, consensus conferences, and the publication of official EU methodological framework.
- *Integrating sustainability into dietary guidance:* (1) applying quantitative modeling that integrates environmental criteria alongside nutritional adequacy, as already implemented in the Netherlands and in Germany; (2) Promote plant-based foods, reduce red and processed meat consumption, and encourage local, seasonal, and minimally processed foods; (3) align dietary guidelines with EU strategies, including climate mitigation and biodiversity protection. Integration could be driven by mandatory environmental criteria embedded in the guideline revision process ensuring that environmental considerations are treated as core parameters alongside nutritional adequacy. This would require the development of standardized methodologies to assess environmental impacts, supported by pilot initiatives and multi-stakeholder collaborations that test feasibility and scalability in different contexts.
- *Strengthen institutional capacity:* (1) support cross-sectoral partnerships to share expertise, reduce duplication of effort, and maintain up-to-date nutrient data; (2) establish multidisciplinary advisory panels to review modeling assumptions, validate data outputs, and refine recommendations before each guideline update; (3) develop interactive platforms that merge dietary intake and FCDB data; (4) use the EFSA portal to publish standardized indicators on survey coverage, database completeness, and FBDGs alignment, enabling policymakers to benchmark performance and adopt best practices. Implementation could be facilitated through mechanisms that foster collaboration and transparency, such as joint initiatives to share expertise and information, a European Hub under EFSA coordination to generate current nutrient intake estimates, monitor adherence to FBDGs, and detect emerging nutrient deficiencies.

## 4 Discussion

The assessment of dietary intake data and the methods used to develop and present FBDGs across the 11 EU countries participating in the PLAN’EAT project highlights substantial differences and inconsistencies. These discrepancies pose significant challenges to the development of coherent and effective nutrition policies at the European level. The findings that came out from the present analysis showed that cultural and structural diversity in Europe represent an obstacle to implementing recommendations. Differences in administrative structures, available resources, traditional dietary habits, and public health infrastructures may influence the feasibility and effectiveness of a harmonized monitoring system.

Despite this, across Europe, the EU Menu project of the EFSA has aimed to harmonize dietary monitoring systems. Other harmonization approaches were also proposed by the studies carried out at the European level by Bel-Serrat and colleagues on surveillance systems for dietary, physical activity and sedentary behaviors (109) and by Hebestreit and colleagues on surveillance of obesity-related lifestyle behaviors (110). Starting with these harmonization proposals and considering the results of the present review, it was clear that further methodological improvements can be achieved. Notable gaps also persist in the management of FCDBs and in the degree of alignment among national FBDGs. Addressing these issues will require coordinated strategies across the EU.

With regard to food consumption data, EFSA has invested considerable effort in harmonizing data collection and dietary assessment methodologies across Member States (30). However, as reported by Mertens et al. (111), variations remain in dietary assessment methods, interview administration, portion size estimation, and dietary software used. These differences affect data comparability and undermine the ability to conduct consistent cross-country analyses.

To enable continuous and systematic monitoring of dietary patterns across Europe, national dietary surveys should be conducted at regular intervals, using harmonized methodology as suggested by EFSA, as it was set up by the new National Nutrition Monitoring programe of Germany established until 2034 (112). Moreover, as Rippin et al. (113) emphasize, heterogeneity in age groups, dietary assessment methods, nutrient composition databases and under-reporting represent barriers to country comparisons. Therefore, harmonizing these aspects for national dietary surveys should be a priority. Such alignment is essential as food consumption data are fundamental to evaluating the effectiveness of food policies and nutrition interventions.

In this context, prioritizing the application of the EU Menu methodology, with regular updates and standardized reporting, is critical. To overcome financial and capacity disparities between countries, one key actionable recommendation is to provide monetary and/or technical support at the EU level for countries with outdated or incomplete dietary surveys, enabling them to conduct methodological consistent assessments. As highlighted by Micha et al. (114), it is important to challenge the prevailing perception that dietary intake data collection is prohibitively expensive or overly complex, given its central role in addressing public health challenges.

Food consumption data are also essential for monitoring and evaluating population adherence to FBDGs. A practical approach is to compare actual dietary intake data with national recommendations at defined intervals after guidelines implementation (115). This approach enables a systematic assessment of adherence to dietary guidelines and the identification of gaps which can inform adjustments policies and interventions aimed at improving FBDG adherence.

FBDG adherence indicators—essentially diet quality indices—are valuable tools in this process, as they assess how closely population-level eating habits aligns with dietary recommendations. These indicators have already been developed and used in various countries within and beyond Europe, including Norway, Italy, France, the Netherlands, Sweden, United States, and Australia, (82, 116–121). They can help identify discrepancies between actual consumption and recommended targets. However, if food consumption data are not considered when updating FBDGs, it becomes challenging to monitor dietary trends, implement region-specific changes, or evaluate the impact of dietary recommendations. Incorporating consumption data ensures that updates are better aligned with actual dietary patterns, making recommendations more realistic and achievable (a strategy already adopted in the Netherlands, Germany, France, Denmark, and the United Kingdom) (59–62, 122, 123). Moreover, indicators measuring adherence to guidelines could be matched with those collected and produced by EU countries and the European Commission and included in the European Core Health Indicators Set (124). Examples include fruit and vegetable consumption, hazardous alcohol consumption, and physical activity levels. In addition, according to the purpose of the monitoring strategy, further selections could be made by consulting scientific works focusing on different types of indicators, such as policy or behavioral indicators concerning dietary habits and physical activity (125, 126). These actions will explore a wide range of public health conditions and therefore help to establish a more solid, well-structured monitoring system. A key objective of the present work is to promote the harmonization of dietary assessment tools and FBDGs across Europe to improve data consistency and comparability. Such harmonization is essential for the development of more effective, coherent, and sustainable food and nutrition policies at both national and EU levels. The comparative analysis in the PLAN’EAT project identifies several challenges and opportunities in this regard. Aligning methodologies, definitions, and nutritional messages can be pursued while respecting national contexts, drawing on shared wellbeing priorities to create a more integrated and responsive policy framework.

Methodologically, countries such as France, Germany, and the Netherlands have implemented dietary modeling systems that combine health and/or environmental criteria with national population food consumption data. This integrated approach provides a more comprehensive evidence base for policy, ensuring recommendations meet both nutritional needs and sustainability objectives. Expanding such modeling approaches across Europe would help ensure that national FBDGs foster healthier and more sustainable food systems (57, 127).

Regarding dietary recommendations, target populations, and behavioral guidance (e.g., physical activity or sleep), there is a broad alignment of core principles across countries. Nevertheless, differences remain in the content, scope, and level of detail reflecting varied national priorities, cultural contexts, and implementation strategies. Discrepancies in quantitative and qualitative recommendations, together with different food group classifications, hinder both clarity of consumer messaging and comparability of guidelines across Member States, and consequently the comparison of the adherence to these guidelines result difficult to achieve Integration of sustainability into FBDG is another aspect to consider. While some countries have incorporated sustainability through explicit recommendations or modeling approaches, others have yet to address it. Promoting reduced red meat consumption and greater adoption of plant-based foods is essential, as these components remain underrepresented in many national and international FBDGs (107, 128).

Equally important is tailoring FBDGs to meet the needs of diverse population groups, from children to older adults, and accounting for physiological conditions and socio-economic diversity (129). The harmonized methodologies for the dietary guidelines proposed in this study could serve as a core EU framework, allowing Member States to adapt and expand upon them to reflect their unique public health contexts and cultural traditions.

This study has several strengths and limitations. A key strength is its broad comparative scope, covering 11 countries from different European macro-regions, which enhances the relevance of findings for European-level policymaking. Another strength is the translation of technical findings into actionable policy recommendations, bridging research and decision-making. The study identifies methodological and content inconsistencies in dietary assessment and FBDGs, emphasizing the need for harmonization. Also, the findings of the PLAN’EAT project could complement and extend the World Cancer Research Found’s NOURISHING framework (130) by identifying specific gaps and opportunities in the implementation of national dietary data systems and FBDGs, especially in relation to methodological harmonization, integration of sustainability principles, and cross-country comparability. By addressing these aspects, the PLAN’EAT approach can support the enhancement and practical application of the NOURISHING framework within the European context.

Moreover, by showcasing successful approaches, such as modeling tools used in France, Germany, and the Netherlands, the work findings support knowledge transfer and the scaling-up of integrated dietary strategies that address health, environmental, and social dimensions in line with the PLAN’EAT project’s objectives.

The main limitation lies in the qualitative nature of the analysis: while it offers detailed insights, it does not include statistical comparisons or quantitative metrics of discrepancies between countries. In addition, the study would benefit from data and input from a wider range of stakeholders, including government bodies, academia, NGOs, consumer organizations to better inform the policy harmonization process. Moreover, this research is limited to a small number of European countries, therefore expanding geographical scope beyond the 11 PLAN’EAT countries could capture a more complete picture of dietary policy diversity in the EU. Finally, while harmonization is both desirable and feasible in many contexts, further research should consider how structural and cultural differences between countries may affect the practical implementation of common and shared recommendations. For instance, the protocol adopted by Hebestreit and collaborators to find a common indicator for monitoring fruit and vegetable consumption across Europe can be used as a reference for further harmonization processes for other food groups (131).

Although significant initiatives, such as EFSA’s EU Menu, have laid important groundwork, substantial disparities in methodologies, data quality, and guideline structure still limit the effectiveness of cross-country comparisons and policy alignments. Strengthening the comparability of data and ensuring methodological consistency will not only enhance the monitoring and evaluation of national and EU-level nutrition policies but also support the development of more sustainable, inclusive, and evidence-based nutritional recommendations. Promoting the integration of sustainability considerations, cultural relevance, and life-stage-specific guidance into FBDGs, while ensuring periodic revisions that align with evolving scientific and societal needs will be crucial to fostering healthier diets and more resilient food systems across Europe. Ultimately, these efforts will empower policymakers, health professionals, and citizens to make informed choices that benefit both population and planetary healthiness.

To conclude, the findings of this study align with and complement other European initiatives aimed at promoting healthier and more sustainable nutrition environments at the system level. In particular, they contribute to the broader evidence base developed by frameworks such as INFORMAS (International Network for Food and Obesity/NCDs Research, Monitoring and Action Support) (132), which monitors food environments and policies across countries. By identifying gaps in dietary assessment, food composition databases, and FBDGs, the present analysis provides actionable insights to strengthen data harmonization and policy coherence within Europe—key objectives shared with similar EU-funded projects such as the Joint Action on Implementation of Validated Best Practices in Nutrition (Best-ReMaP) (133) and the Joint Action Prevent Non-Communicable Diseases (PreventNCD) (134). Integrating the PLAN’EAT findings with these system level initiatives can thus help advance coordinated efforts toward healthier, more equitable, and sustainable food environments across the EU.

## Footnotes

1
https://planeat-project.eu
