# Correction: Dietary assessment and dietary guidelines across 11 European Union countries: a review from the PLAN'EAT project

**DOI:** 10.3389/fnut.2026.1793148

**Published:** 2026-02-03

**Authors:** Vittoria Aureli, Federica Grant, Alicia Aguilar-Martínez, Anke Brons, Anthony Fardet, Betty Chang, Francesca Vespa, Jana Kirschner, Ewa Kopczynska, Lajos Böröcz, Maria Jacobsen, Siranush Ghukasyan, Yannis Manios, Wencke Gwozdz, Emese Antal, Laura Rossi

**Affiliations:** 1 Departmental Faculty of Science and Technology for Sustainable Development and One Health, University Campus Bio-Medico of Rome, Rome, Italy; 2 Council for Agricultural Research and Economics - Research Centre for Food and Nutrition (CREA - Food and Nutrition), Rome, Italy; 3 Department of Agricultural, Forest and Food Sciences (DISAFA), University of Turin, Grugliasco, Italy; 4 Department of Agricultural, Environmental and Food Sciences (DiAAA), University of Molise, Campobasso, Italy; 5 Epi4Health Research Group, Faculty of Health Sciences, Universitat Oberta de Catalunya, Barcelona, Spain; 6 Public Administration and Policy, Department of Social Sciences, Wageningen University, Wageningen, Netherlands; 7 INRAE, Clermont-Auvergne University, Unité de Nutrition Humaine, Clermont-Ferrand, France; 8 European Food Information Council, Brussels, Belgium; 9 Division of Bioeconomics, Department of Earth and Environmental Sciences, Katholieke Universiteit Leuven, bLeuven, Belgium; 10 European Public Health Alliance, Brussels, Belgium; 11 Institute of Sociology, Jagiellonian University, Kraków, Poland; 12 Hungarian Hospitality Employers' Association (VIMOZS), Budapest, Hungary; 13 Department of Energy and Technology, Swedish University of Agricultural Sciences, Uppsala, Sweden; 14 TMG Think Tank for Sustainability, Berlin, Germany; 15 Department of Nutrition and Dietetics, School of Health Science and Education, Harokopio University, Athens, Greece; 16 Hellenic Mediterranean University Research Center, Institute of Agri-Food and Life Sciences, Heraklion, Greece; 17 Department of Consumer Behaviour, Nutritional Communication and Sociology, Faculty of Agricultural Sciences, Nutritional Sciences and Environmental Management, Justus-Liebig-University, Giessen, Germany; 18 Department of Management, Society and Communication, Copenhagen Business School, Copenhagen, Denmark; 19 Environmental Social Science Research Group, Budapest, Hungary; 20 Health Sciences Division, Doctoral College, Semmelweis University, Budapest, Hungary; 21 Department of Food Safety, Nutrition, and Veterinary Public Health, National Institute of Health, Rome, Italy

**Keywords:** nutritional policies, public health, sustainability, harmonized approaches, PLAN'EAT project, Europe

The **reference 62** was erroneously written as Schäfer A, Boeing H, Gazan R, Conrad J, Gedrich K, Breidenassel C, et al. A methodological framework for deriving the German food-based dietary guidelines 2024: food groups, nutrient goals, and objective functions. medRxiv [Preprint]. (2024). doi: 10.1101/2024.10.24.24316069. It should be Schäfer AC, Boeing H, Gazan R, Conrad J, Gedrich K, Breidenassel C, et al. A methodological framework for deriving the German food-based dietary guidelines 2024: food groups, nutrient goals, and objective functions. *PLoS ONE*. (2025) 20:e0313347. doi: 10.1371/journal.pone.0313347

There was a mistake in Table 3 as published. The responsible body that made FBDGs for Germany and the wording for the FBDGs for Germany are not correct. The corrected Table 3 appears below.

**Table 3:** List of national FBDGs of European countries participating in the PLAN'EAT project.

**Country**	**Food-Based Dietary Guidelines**	**Responsible body**	**Update**	**References**
Belgium	Belgian dietary guidelines	Superior Health Council	2019	(63)
France	French dietary guidelines, PNNS 4 (the Programme National Nutrition Santé n°4)	National Agency for Food, Environmental and Occupational Health and Safety (ANSES)	2019	(64)
Germany	Food-Based Dietary Guidelines for Germany	German Nutrition Society (DGE)	2024	(65,66)
Greece	The “National Nutrition Guide for Greek” (NDGGR)	Representatives of academia, Ministry of Health and Ministry of Education and Culture	2017	(67)
Hungary	SmartPlate	Hungarian Dietetic Association and the Hungarian Academy of Sciences	2021	(68)
Ireland	The Healthy Eating Guidelines	Department of Health (Health Service Executive)	2016	(69)
Italy	The Italian dietary guidelines	CREA-Food and Nutrition Research Center	2018	(70)
The Netherlands	Wheel of Five	Netherlands Nutrition Centre (NNC)	2024	(58, 71)
Poland	Plate of Healthy Eating	National Institute of Public Health PSH—National Research Institute—National Institute of Hygiene (NIZP-PZH NIZP PZH–PIB	2021	(72, 73)
Spain	Spanish dietary guidelines	Agencia Española Seguridad Alimentaria y Nutrición (AESAN)	2022	(74)
Sweden	Find your way—Eating habits and dietary guidelines	Swedish National Food Agency (Livsmedelsverket)	2025	(75)

There was a mistake in Table 4 as published. The FBDG recommendations for Germany were not updated according to the last releases. The corrected table 4 appears below.

**Table 4:** FBDGs recommendations for common food groups targeting the adult population in the European countries participating in the PLAN'EAT project.

**Food groups**	**Belgium**	**France**	**Germany**	**Greece**	**Hungary**	**Ireland**	**Italy**	**The Netherlands**	**Poland**	**Spain**	**Sweden**
**Fruit and vegetables**	250 g of fruit and 300 g of vegetables/day	5 fruit and vegetables/day	5 potions/day SP: 110 g	3 servings of fruit and 4 servings of vegetables/day SP vegetables: 150–200 g raw/cooked and fruit: 120–200 g	At least 5 portions/day: 3–4 portions of vegetables/1–2 portions of fruit (at least 1 portion should be fresh/freshly cut) SP: 1 large pepper, tomato, 1 large apple or peach or 1 medium bowl of lettuce or 80 g dry or 120 g fresh/frozen pulses or 1 cup of berries or 2 dl smoothie	5–7 servings/day SP: 1 medium size fruit, 2 small fruits, $\frac{1}{2}$ cup cooked vegetables, 1 bowl salad	3 of fruit and 2 $\frac{1}{2}$ of vegetables times/day SP fruit: 150 g and vegetables: 200 g	250 g of vegetables and 200 g of fruit/day SP vegetables: 50 g and fruit: 100 g	400 g/day $\frac{1}{4}$ of plate is fruit; $\frac{3}{4}$ of plate is vegetables	3 servings of fruit at least and 2 servings of vegetables/day SP vegetables: 150–200 g and fruit: 120–200 g	500 g/day vegetables, fruit and berries
**Legumes**	Included in the vegetables consumption Consume legumes every week	At least twice a week; can replace meat but to be combined with cereals	1 portion/week SP: 125 g (ready to eat)	3 servings/week SP: 150–200 g of cooked legumes	At least once a week (included in the vegetables consumption)	2 servings/day^*^ SP: $\frac{3}{4}$ cups	3 times/week SP: dry legumes: 50 g; fresh legumes: 150 g	2–3 servings/week SP: 60 g	2–3 times/week SP: 50 g; dry portion	4 servings/week SP: 50–60 g raw	Eat beans, peas and lentils often—preferably every day.
**Nuts**	15–25 g/day	A handful/day	1 portion/day (together with seeds) SP:25 g	1–2 servings/day SP: 18 almonds, 6 whole walnuts, 3 tablespoons of sunflower seeds	2–3 times/week SP: small handfuls of nuts, unsalted almonds, hazelnuts, oilseeds such as pumpkin seeds	2 servings/day^*^ SP: 40 g	2 times/week SP: 30 g	15–25 g/day	30/40 g/day	3 or more servings/week SP: 20–30 g	Eat two to three tablespoons of unsalted nuts a day
**Grain-based foods Whole grains**	At least 125 g/day of whole-grains	At least one portion of whole-grain starchy food/day	5 portions/day SP:60 g (one slice of bread, one serving of cereal flakes, uncooked pasta/rice) At least 1/3 should be whole grain	5–8 serving of refined and whole-grain cereals/day SP: 1 slice or 30 gr of bread, 1/2 cup of cooked pasta or rice, 1/2 of breakfast cereals, 1 medium potato: 120–150 g cooked	3 times/day at least one portion out of three should be whole-grain SP: 1 piece of sweet bread dough or 1 medium slice bread/brioche bread or 12 tablespoons (200 g) cooked pasta/rice or 3 tablespoons of breakfast cereals	3–5 servings/day SP: 1 cup cooked rice, pasta, noodles or cous cous, 2 thin slices whole meal bread Enjoy whole grains at each meal	Bread 3 ^1/2^ times/day SP 50 g; Pasta, rice, etc. 1 ^1/2^ times/day SP: 80 g Prefer whole-grain products	Bread 4–8 slides/day; SP: 35 g 3–5 servings/day of cereal products and potatoes; SP: tablespoon of cereals: 50 g; medium potato: 70 g At least half of whole-grain grain products every week	90 g 3 times/day of whole grain cereals	3–6 servings/day SP: 40–60 g bread, 60–80 g pasta, rice Prefer whole-grain products	At least 90 grams of whole grains per day
**Meat Red meat Processed meat White meat**	Maximum 300 g/week of red meat Maximum 30 g/week of processed meat 1–3 times/week (including eggs/meat substitutes) Limit the consumption of red meat, especially processed meat. Red meat can be replaced by e.g. legumes, fish, eggs or poultry	Prioritize poultry and limit red meat to 500 g/week Charcuterie: limit to 150 g/week	1–2 portions of beef, pork, poultry/week SP: 120 g 2 portions of sausages/week SP:30 g Do not consume more than 300 g/week of meat and sausage	1 serving of lean red meat/week SP: 120–150 g of cooked meat 1–2 servings of white meat/week SP: 120–150 g of cooked meat. Processed meat: as few as possible.	Choose lean variants more often. Consume not more than 350–500 g/week of cooked/steamed/fried red meat (e.g. beef, pork). Processed meat only occasionally, in small amounts.	2 servings/day^*^ SP: 50–75 g (beef, lamb, pork, poultry) Limit processed salty meats	Once/week of red meat SP: 100 g 2 times/week of white meat SP: 100 g Limit the consumption of processed meat (occasionally consumption)	Max 500 g/week of which max 300 g of red meat SP: 100 g/day excluding processed meat and eggs Limit the consumption of red and processed meat.	Not more than 350–500 g of red meat and processed meat/week For the white meat: choose lean poultry meat (e.g. chicken, turkey) without the skin	0–3 servings/week for meat, preferring the white meat SP:100-125 g For processed meat: reduce or even avoid consumption	No more than 400–500 g of raw meat. (no more than 350 g cooked) Only a small part of it should be cured meat.
**Fish**	1–2 times/week (oily fish once/week)	2 times/week of which once fatty fish	1–2 portions/week SP: 120 g	2–3 servings of fish and seafood/week SP: 150 g of cooked fish or seafood	At least once/week Prefer local fish (e.g. trout, catfish, bighead carp).	2 servings/day ^*^ SP: 100 g	2 times/week SP: 150 g	1 serving/week, preferably fatty fish SP:100 g	2 times/week SP: 100–150 g	3 servings/week SP: 125–150 g	2–3 times/week
**Dairy products Milk Yogurt Cheese**	250–500 ml/day of dairy	2 times/day of dairy	2 portions/day Milk SP: 250 g Yogurt SP: 150 g Cheese SP: 30 g	2 servings/day SP: 250 ml of milk, 200 g of yogurt, 30 g of seasoned cheese, 60 g of fresh cheese	Every day SP: 200 ml milk/yoghurt/kefir or 50 g cottage cheese or 30 g cheese	3 servings/day SP: 200 ml of milk, 125 g of yogurt, 25 g of cheese	Milk/yogurt 3 times/day SP: 125 ml/125 g Cheese 3 times/day SP:100 g fresh; 30 g seasoned	2–4 servings/day of milk and dairy SP:150 g Cheese SP: 40 g/day	Prefer low-fat products	3 servings/day SP: milk: 200–250 ml; fresh cheese 85–125 g; seasoned cheese 40–60 g; 125 g yogurt	Milk and yogurt every day; cheese can replace a small share of milk; refer low fat products.
**Eggs**	1–3 times/week with poultry/meat substitutes	NA	1 egg/week SP:60 g	At least 4 times/week	Replace meat with other protein sources, including eggs	2 servings/day^*^	3 times/week SP: 50 g	2–3 times/week SP: 50 g	Good source of proteins and other nutrients, maximum 7 eggs a week	4 times/week SP: 50–60 g	Good alternative to meat
**Sugary foods**	Consume as few drinks with added sugars as possible and choose water instead	Limit the foods rich in sugar	Avoid products with sugar	Limit added-sugar products/sugar	Limit added-sugar products/sugar	NOT every day	Occasionally	Outside the wheel of five	Reduce the consumption	Avoid the consumption	Limit sugary products
**Oils/fats**	Prefer non-tropical oils, spreadable fats and soft or liquid cooking fats	Prefer olive, walnut, rapeseed oil	1 tablespoon/day of vegetable oils 1 tablespoon/day of butter/margarine SP: 10 g	Prefer olive oil (4–5) times/day; SP: 15 ml	Less fat for cooking, prefer oils	Rapeseed, olive, canola, sunflower or corn oils (in a very small amount)	3 times/day SP: 10 ml	Spreadable and cooking fats (not specified) SP: 35–65 g	Prefer vegetable oils, reduce animal fat	Olive oil in every meal (10 ml)	Prefer vegetable oils

The original version of this article has been updated.

