# Supplementary material for: Dietary assessment and dietary guidelines across 11 European Union countries: a review from the PLAN’EAT project
Source: Front Nutr. 2026 Jan 12;12:1699036. doi: 10.3389/fnut.2025.1699036 (PMC12832486; doi:10.3389/fnut.2025.1699036)
Supplement: Supplementary file 1 [file Table_1.pdf]

Supplementary Material

**Data and Information required**
Country page template: please indicate your Country ______
**Items to be collected in all Territories**<br><br>The focus of this template is on gathering data and information regarding four main topics:<br>food composition database, dietary guidelines, food consumption and food behaviors.<br>Please fill in the boxes below providing information that comes from scientific papers, reports and projects, grey literature, including websites and documents in your local language.<br>Please, take in mind that each information added in the template must have a valid reference.<br>Please add each citation in the text and the bibliography at the end of the document.<br>Other clarifications:<br>- Having in mind that the subject of the survey is "your Country". Data and information need to be collected from experts and representatives of each sectors investigated.<br>- Answer such as "do not know", "not studied yet", "not evaluated yet" ect. are possible; in this case provide an explanation of the absence of information.
**FOOD COMPOSITION DATABASE**
1. **Food composition database: main contents**
1.1.Does your Country have a national food composition database?
☐ Yes ☐ No
If yes, could you describe it? When did the first version come out? When was the last version released? Do you have a specific website which collects your Country food composition database?
(Please, provide a description of the last version of the database specifying how many food items it contains and which components are reported, e.g. carbohydrates, proteins, fats, vitamins).
If no, what database does your Country usually use?
(Please provide a brief description of this database).

1.2.Are data, that your Country use, freely downloadable by everyone?
(Please answer to the following questions (from 2 to 7) also if your Country does not have its own database-both private and public).
2. **Regulation for the creation of the food composition database**
2.1. Did your Country follow specific recommendations to create the database?
☐ Yes ☐ No
If yes, provide a quick overview of the process
(Please provide an overview of the process and interventions made by any organizations - both public and private - that led the creation of the database).
If no, which scheme/procedure/guidelines did your Country follow?
(Please provide an overview of the process and interventions made by both political and private organizations that led the creation of the database).
3. **Techniques to create the food composition database**
3.1. Who was responsible for the creation of the database?
(Please provide a description of the type and the role of the experts that participated in this process)
3.2. What kinds of methodologies were used to build up the database?
(Please provide an overview of the methodologies/protocols/tools used and highlight their limits and strengths.)
**1. Any weaknesses about the database**
4.1. Are there any weaknesses about the database?
☐ Yes ☐ No
If yes, what kind of weaknesses? Are your Country working to solve them?
(Please provide a specific list of what kind of components of the database could be improved and the reason).
4.2. Are there any strengths of the database?
(Please provide a description of the main characteristics that make the database a useful tool for your Country research).
**2. Food sustainability**
5.1. Is there any information regarding the environmental sustainability of a product?
☐ Yes ☐ No
If yes, what kind of information?
(Please provide a description of indicators or simple information on this topic).

If no, would you like to add this kind of information to the database? Do you already have plans for this addition?
(Please provide a brief description of the plan or of the idea that you would apply).<br><br>*We have not yet contacted the Max Rubner Institute in this regard.*
3. **Future plans**
6.1. Are there any plans to update the current database??
☐ Yes ☐ No
If yes, which kinds of changes are your Country going to make? Will the environmental sustainability aspects be improved?
(Please provide a description of which are the future changes and the reasons which brought you to make them, explaining in depth what changes will be made to environmental sustainability aspects (if there will be any)).
If no, are your Country planning to realize modifications in the future?
Please provide a brief description of ideas/plans/intentions for the future.
**4. INFOODS**<br><br>INFOODS is the International Network of Food Data Systems that, with FAO, provides guidelines, standards, compilation tools, databases, capacity development tools, policy advice, advocacy tools, technical assistance at country level.
7.1. Are you aware of the INFOODS network?
☐ Yes ☐ No
If yes, is your Country a partner?
(Please provide a brief description of your Country role in this network).
If no, why? Would you like to become a partner?

**DIETARY GUIDELINES**
1. **National dietary guidelines**
1.1.Do you have any national dietary guidelines in your Country?
☐ Yes ☐ No
If yes, how long have they been published? Who was responsible for their creation? How often and by who are they updated?<br><br>The nutrition recommendations have been published by the German Nutrition Society since 1950. Reference values have been updated approx. annually since then.

1.1.1. What kind of methodologies were used to realize them and how are they structured?
1.1.2. What are the major topics of your Country's guidelines?
(Please, provide a concise description of the major topics of your Country's guidelines, including recommendations for specific groups (e.g., children and adolescents, pregnant women, older people…), if there are any).
If no, why? Do you have any plans to publish them in the future?
(Please, provide a brief description of your Country's plans to publish dietary guidelines in the future).
2. **Food sustainability issues**
2.1. Do you have any reference to sustainability (environmental, social, economic) in your guidelines?
☐ Yes ☐ No
If yes, could you describe it?
(Please, provide a concise description of the major sustainability topics of your Country's guidelines).
If no, do you have any plans to add this topic in the future?
(*Please, provide a brief description if your Country has plans to add guidelines about sustainability in the future*).
3. **Behavioral advice**
3.1. In addition to dietary recommendations, is there also behavioral advice? (e.g. physical activities, smoking, breakfast...)
☐ Yes ☐ No
If yes, could you describe them?
(*Please, provide a concise description of the major behavioral advice of your Country's guidelines).*
If no, why? Do you have any plans to add this section in the future?
(Please, provide a brief description if your Country has any plans to add this section in the future).
4. **Changes in eating habits**
4.1. Has there been an improvement in the eating habits of the population after the guideline's publication and its updates? (e.g., increased consumption of fruits and vegetables, increased consumption of legumes, reduction of sugar, fruit juices, fats, red meat, etc..).
☐ Yes ☐ No

If yes, please provide the list of the possible improvements that happened after the guidelines' publication together with an overview of studies that analyzed changes and the methodology used for the evaluation).
5. **Communication activities**
5.1. Does your Country organize communication activities towards the population to spread the dietary guidelines? By whom are they carried out?
☐ Yes ☐ No
(If yes, please provide an overview of all activities performed, including school programs, conferences, online video summaries etc..).
6. **Limitations and future prospective**
6.1. Are there any limitations in the dietary guidelines of your Country? (e.g. behavioral changes difficult to achieve/poor compliance; lacking of some topics; difficulties with the communication/technicality in the wording; absence of a graphical form etc.)
☐ Yes ☐ No
If yes, could you describe it? Do you have any plans to overcome them?
(*Please, provide a written account of a concise description of the major limitations of your Country's guidelines).*
**FOOD CONSUMPTION**
1. **Food consumption**
1.1. Does your Country assess the population food consumption?
☐ Yes ☐ No
If yes, can you provide a description?
(Please provide information regarding who is responsible for the research, what is the methodology applied and how food consumption and diet composition is calculated, and which population groups are included).
If no, is your Country planning to set up a study?
(Please provide a description of the study that you are going to carry out).
2. **Trends**
2.1. Which changes did you observe in food consumption in the last 10 years (e.g. an increase or a decrease in a particular food group)
(Please provide a list of the food group changes and the possible economic, social and environmental impacts).

3. **Food consumption dataset**
3.1.Does your Country have a dataset for food consumption?
☐ Yes ☐ No
If yes, could you describe it?
(Please provide information regarding who is responsible for its creation (specifying the type and the role of the experts), when it was realized, when is the last update, how is structured, if it has a private or public access).
3.2.Does the dataset have some shortcomings?
If yes, what are these shortcomings?
(Please provide a description of the shortcomings and if you are adopting strategies to solve them).
If no, could you underly the main strength of the dataset?
4. **Food consumption and recommendations**
4.1.Please provide an overview of the relation between food consumption and what your Country dietary guidelines recommend, highlighting what is in line.
4.2.Please provide an overview of the relation between food consumption and what your Country dietary guidelines recommend, highlighting what is not in line.
5. **Sustainable consumption**
5.1.Has your Country studied food consumption with regard to sustainability? (e.g. focusing on the economic, environmental and social sustainable aspects).
☐ Yes ☐ No
If yes, could you describe how your Country conducted the study?
(Please provide the description of the research methodology and the main findings).
If no, are you going to carry out this study in the future?
(Please provide the idea of the study project).
6. **Policy actions to encourage the shift towards sustainable consumption**
6.1. Has your Country established actions (e.g. educational program, awareness campaign, food labelling, taxes, educational policy such as school meals or food curriculum, restaurant and hotel food requirements, etc..) to lead the transition towards more sustainable consumption?
☐ Yes ☐ No
If yes, could you describe them?

(Please provide details regarding main laws and specify if there is a determined action plan, describing it, in particular listing existing articles / documents / reports which collects these information).
If no, are you aware of any policies or strategies to adopt as measures to move towards this objective that your government is preparing?
6.2. Is your Country adopting actions to achieve one or more specific targets of the 12 SDG regarding sustainable consumption and production?
If yes, what kind of actions? (e.g. project, research actions, educational programs, awareness campaigns, etc...)
(Please provide a description of the target on which your Country is working (e.g. food waste, sustainable food consumption, sustainability reports, sustainable public procurement policy, etc...) and then the actions (e.g. project and data collection, broad research, educational programs, awareness campaigns) towards the achievement of the 12 Goal; provide also a description of indicators of the progress of these actions, if there are any).
If no, could you explain the reason?
**EATING HABITS**
5. **Eating habits and cultural diets**
1.1. Could you describe the eating habits and cultural diets of your Country general population?
Please, provide a description of the major eating habits (omnivorous, flexitarian, vegetarian...) and cultural diets (Mediterranean, Western...) of your country. Take into consideration ethnic groups in your regions.
1.2. How have the population's eating habits changed in the last 10 years?<br><br>Please provide a brief description of the changes in eating habits that may have occurred in your Country (e.g. increasing in the number of vegetarians and/or vegans, eating more plant-based food or increasing the consumption of animal products, more attention towards food waste, attention to locally produced foods, etc.).
1.3. Have any shifts towards more environmentally sustainable eating habits and diets been evaluated?
☐ Yes ☐ No
If yes, please provide an overview of studies/reports that analyzed changes and the methodology used for the evaluation.
1.4. What is the percentage of the population in your Country that has food allergies or intolerances (e.g. celiac disease, lactose intolerance, nickel allergy...)?
1.4.1. How has the consumption of "*free*" products changed in the last 10 years? (e.g. gluten-free, lactose-free, etc...)
6. **Drivers**
2.1. Did your Country identify specific drivers that led food consumption in your country? (i.e. socio-economic factors, environmental issue, marketing strategies, policy actions, recommendations, guidelines, etc...)
☐ Yes ☐ No
If yes, please provide a list of these drivers and the how they were identified (e.g. surveys, literature studies)

7. **Population's meal daily pattern**
3.1. What is the meal daily pattern of your Country general population? (E.g. 3 meals/day, 5 meals/day..)
(Please, provide a description of the typical daily pattern of your country, specifying if there are meals (breakfast, lunch and dinner) and/or snacks).
**8. Macronutrients daily proportion**
4.1. What is the macronutrients proportion (carbohydrates, fat and proteins) recommended in your Country?
**9. Plant-based food**
5.1. How many servings of fruit and vegetables per day are recommended in your Country? What is the recommended standard portion (g/day)?
5.2. Do your Country recommend the consumption of seasonal fruit and vegetables?
5.3. Are there any government projects in your Country to encourage the consumption of fruit and vegetables?
☐ Yes ☐ No
If yes, please provide a description of the government's main interventions.
5.4. How many servings of legumes per week are recommended in your Country? What is the recommended standard portion (g/week)?
5.5. Did you notice a change in the subjective attitude/purchasing/consuming of the population towards legumes in the last 10 years in your Country?
☐ Yes ☐ No
If yes, please provide a description of these changes.
5.6. Are there any government projects in your Country to encourage the consumption of legumes?
☐ Yes ☐ No
If yes, please provide a description of the government's main interventions.
5.7. How many servings of nuts per day or week are recommended in your Country? What is the recommended standard portion (g/day or week)?
**10. Grain-based foods**
6.1. How many servings of refined and whole-grain cereals per day or week are recommended in your Country? What is the recommended standard portion (g/day or week)?
**11. Meat**
7.1. How many servings of meat per week are recommended in your Country? And processed meat? What is the recommended standard portion (g/week)?

(Please, take in mind that red meat refers to unprocessed mammalian muscle meat (e.g. beef, veal, pork, lamb) including that which may be minced or frozen; processed meat refers to meat that has been transformed through salting, curing, fermentation, smoking or other processes to enhance flavor or improve preservation and white meat refers to meat that comes from the breast or other thick parts of a chicken, turkey, etc).

7.2. Did you notice a change in the subjective attitude/purchasing/consuming of the population towards meat in the last 10 years in your Country?

☐ Yes ☐ No

If yes, which kind of meat? Was this change related to health, ethic and/or environmental reasons?

7.3. Are there any government projects in your Country aim to reduce the consumption of meat?

☐ Yes ☐ No

If yes, please provide a description of the government's main interventions.

12. **Fish and seafoods**

8.1. How many servings of fish and seafoods per week are recommended in your Country? What is the recommended standard portion (g)?

8.2. Did you notice a change in the subjective/purchasing/consuming attitude of the population towards fish and seafoods in the last 10 years?

8.3. If yes, this change was related to health, ethic and/or environmental reasons?

☐ Yes ☐ No

13. **Fats**

9.1. Which type of fats is the most common used in your Country? (e.g. extra olive oil, olive oil, sunflower oil, peanut oil, rapeseed oil, butter, margarine)

9.2. How are fats mainly used? To season? To cook? To fry?

14. **Breakfast habits**

10.1. What is the percentage of the population that usually have breakfast every morning in your Country?

10.2. What is the most common type of breakfast in your Country?

(P*lease provide a description)*

10.3. Where is it consumed (e.g., home, bar...)?

15. **Alcohol**

11.1. What is the percentage of the population that drinks alcohol in your Country? Do you know the percentage of <18 years that drinks alcohol? What is the most common source of alcohol (e.g. beer, wine, spirits, drinks...)?

11.2. What is the current recommendation for alcohol consumption in your Country? Are there any government projects to reduce the consumption of alcohol?

☐ Yes ☐ No
If yes, please provide a description of the government's main interventions.
16. **Processed and ultra-processed foods**
12.1. What is the definition of processed foods that your Country use? And of ultra-processed foods?
12.2. What is the percentage of processed foods consumed by the population in your Country? And of home-made processed food? (e.g. bread, pizza, desserts, etc..).
12.3. What is the percentage of ultra-processed foods consumed by the population in your Country?
12.4. What are the most consumed ultra-processed foods in your Country?
12.5. Are there any restrictions in your Country for ultra-processed foods?
☐ Yes ☐ No
If yes, please provide a description of the limitations that are currently in place in your Country.
17. **Household dietary diversity score (HDDS)**<br><br>*(The Household Dietary Diversity Score (HDDS) was released in 2006 as part of the FANTA II Project as a population-level indicator of household food access. Household dietary diversity can be described as the number of food groups consumed by a household over a given reference period and is an important indicator of food security).*
13.1. Has your Country calculated the population's HDDS?
☐ Yes ☐ No
If yes, please provide a description of the main results.
